# Spontaneous Hemoperitoneum Due to Ruptured Uterine Artery: A Rare Complication During Pregnancy

**DOI:** 10.7759/cureus.58033

**Published:** 2024-04-11

**Authors:** Rita Palma, Inês Gomes, Catarina Silva, Elisa Pereira, Manuela Almeida

**Affiliations:** 1 Obstetrics and Gynaecology, Hospital Garcia de Orta, Almada, PRT; 2 Gynaecology, Hospital Garcia de Orta, Almada, PRT; 3 Gynaecologic Oncology, Hospital Garcia de Orta, Almada, PRT; 4 Maternal Fetal Medicine, Hospital Garcia de Orta, Almada, PRT

**Keywords:** acute abdomen in pregnancy, third trimester complications, uterine artery rupture, spontaneous hemoperitoneum, hemoperitoneum in pregnancy

## Abstract

Spontaneous rupture of the uterine artery is a rare and life-threatening cause of hemoperitoneum in pregnancy, associated with high maternal and perinatal morbidity and mortality. We present a case of a 29-year-old woman, in the 36th week of gestation, with acute abdomen due to hemoperitoneum. Ultrasound revealed free fluid in the abdominal cavity, with no signs of fetal distress, and the patient was mildly hypotensive. Exploratory laparotomy and cesarean section were performed, and extensive blood clots on the upper abdominal quadrants were discovered, as well as a bleeding left uterine artery. We expect that this case raises awareness of the ruptured uterine artery as a possible etiology of hemoperitoneum during pregnancy.

## Introduction

Hemoperitoneum following spontaneous rupture of uterine vasculature during pregnancy is a rare and emergent condition, with high maternal and perinatal mortality. The clinical presentation may vary from extremely vague symptoms to severe sudden abdominal pain with hemodynamic instability [1-4]. The majority of reported cases occurred in the third trimester of pregnancy [1,3,4], and the diagnosis is mostly done after exploratory laparotomy. When admitting a pregnant patient with acute abdomen, especially with hemodynamic instability, it is of utmost importance to exclude rupture of uterine vessels [3].

## Case presentation

A 29-year-old nulliparous woman at 36 weeks of gestation presented to the emergency department with severe acute abdominal pain with 1 hour of evolution, after a bowel movement, with no history of vaginal bleeding, loss of amniotic fluid, or uterine contractions and no history of abdominal trauma, sexual intercourse, or other physical effort. Relevant past history included a suspicion of endometriosis. On physical examination, the patient was mildly hypotensive, had intense pain on abdominal palpation with muscle guarding, no vaginal bleeding was observed, and uterine tonus was normal; on abdominal and endovaginal ultrasound, there was a considerable amount of perihepatic and perisplenic anechoic free fluid, the cervix was long and closed, there were no signs of placenta praevia or placental abruption, and a single fetus was alive.

Assuming hemoperitoneum in a near-term pregnancy, with considerable hemodynamic stability and a reassuring cardiotocogram, the team decided to perform an exploratory laparotomy under regional anesthesia.

Blood work on admission revealed hemoglobin of 12 g/dL and normal coagulation values.

**Figure 1:**
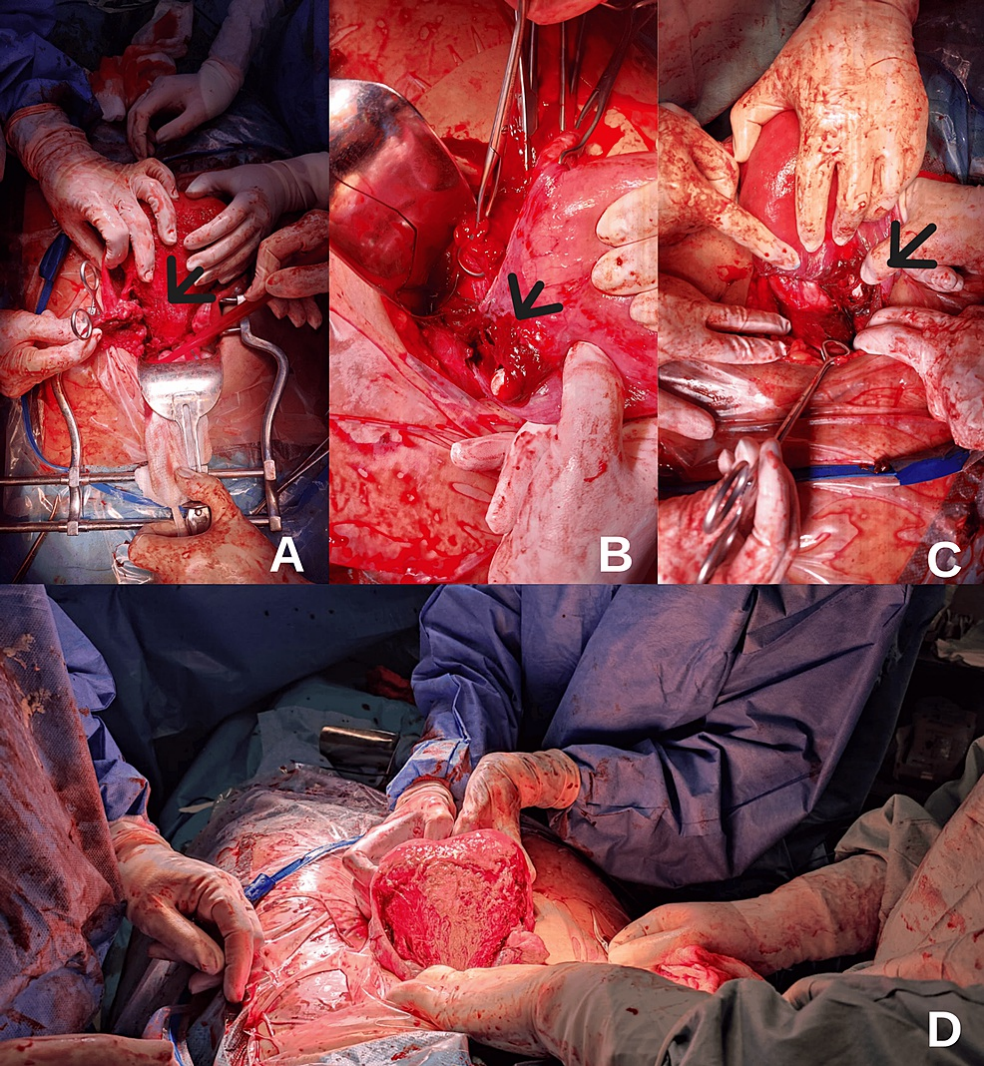
Laceration of uterine artery A,B,C: Laceration extending from the left ovary to the left infundibulopelvic ligament including rupture of a branch of the left uterine artery is visible. D: Extensive decidualization of the posterior uterine wall.

Exploratory laparotomy demonstrated large blood clots in the upper quadrants of the abdomen and Douglas pouch and an intact uterus. A hysterotomy was performed and a girl was born with 2622 g, Apgar score 8/9. There were no signs of placental abruption. Further exploration of the abdominal cavity revealed extensive decidualization of the posterior uterine wall and a bleeding laceration extending from the left ovary to the left infundibulopelvic ligament including a rupture of a branch of the left uterine artery (Figure 1). Adequate hemostasis was achieved by suturing, oxytocin, and tranexamic acid, and sulprostone was administered to further increase uterine contraction. The patient received one unit of packed red blood cells during the procedure. The estimated blood loss during surgery was 900 mL. After adequate hemostasis, the remaining surgery underwent uneventfully. No adnexal masses and no other endometriotic lesions were observed and the uterus was well contracted. Blood test after surgery revealed a decrease of hemoglobin to 8.2 g/dL, which remained stable in subsequent evaluations.

The patient fully recovered and was discharged four days after surgery, with a healthy newborn.

In the follow-up appointment, three months after birth, the patient was asymptomatic and the abdominal incision had healed. Histopathology of the placenta showed chorangiosis foci, and a full workup regarding autoimmunity, thrombophilias, and antiphospholipid syndrome panel was negative.

## Discussion

Spontaneous rupture of uterine vessels is a rare complication, associated with very negative maternal and perinatal outcomes (40% and 30% mortality, respectively) [1,2,4-6]. It may occur in an otherwise uncomplicated pregnancy [2,5].

The etiology may be connected to factors regarding the anatomy of the vasculature, hemodynamic factors, and hormonal levels, although in most cases the exact origin cannot be defined [1-7].

Differential diagnoses include uterine rupture, rupture of adrenal, splenic, kidney, or hepatic veins, congenital malformations, aneurysm rupture, endometriosis, or iatrogenic injuries [1,3-8].

In this case, there was a history of endometriosis, and the symptoms initiated after a strong Valsalva maneuver (perhaps the increased intra-abdominal pressure and consequent increased venous pressure against an already weakened vasculature due to endometriosis motivated the vessel laceration).

There are reports of endometriosis causing hemoperitoneum due to rupture of uterine wall vessels during pregnancy [1,5,8].

Most reported cases refer to the involvement of left-sided uterine vessels, similar to our case, possibly because of the combination of natural dextro-rotation of the uterus during pregnancy, the more common left positions of the fetal head, and the characteristic vessel tortuosity of the pregnant status [2].

The diagnosis is most often made intraoperatively, with aggressive resuscitation and prompt exploratory laparotomy with adequate hemostasis [1-4,7].

## Conclusions

Hemoperitoneum after uterine artery rupture is a serious complication demanding fast recognition and treatment. When a pregnant patient presents with sudden abdominal pain, even if hemodynamically stable, this entity must be excluded or treated accordingly, through immediate fluid replacement and intraoperative hemostasis. A laparotomy with or without cesarean section may be performed depending on the gestational age.
